# Supplementary material for: Emission and Migration of Nanoscale Particles during Osseointegration and Disintegration of Dental Implants in the Clinic and Experiment and the Influence on Cytokine Production
Source: Int J Mol Sci. 2023 Jun 2;24(11):9678. doi: 10.3390/ijms24119678 (PMC10253915; doi:10.3390/ijms24119678)
Supplement: Supplementary file 1 [file ijms-24-09678-s001.zip › ijms-2420122-supplementary.pdf]

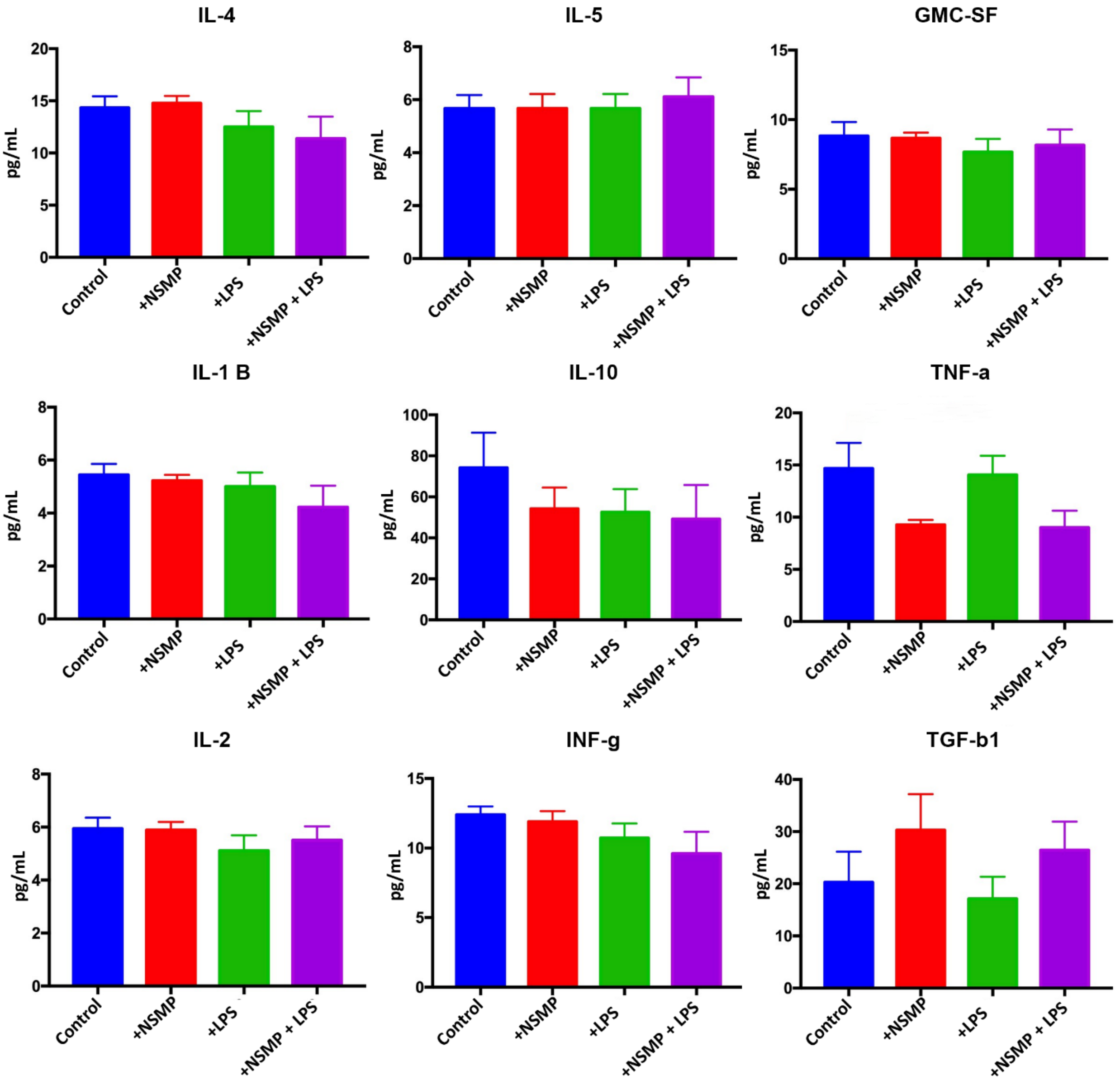

Supplementary Figure S1. Changes in the cytokine cascade when co-cultured with neutrophils.

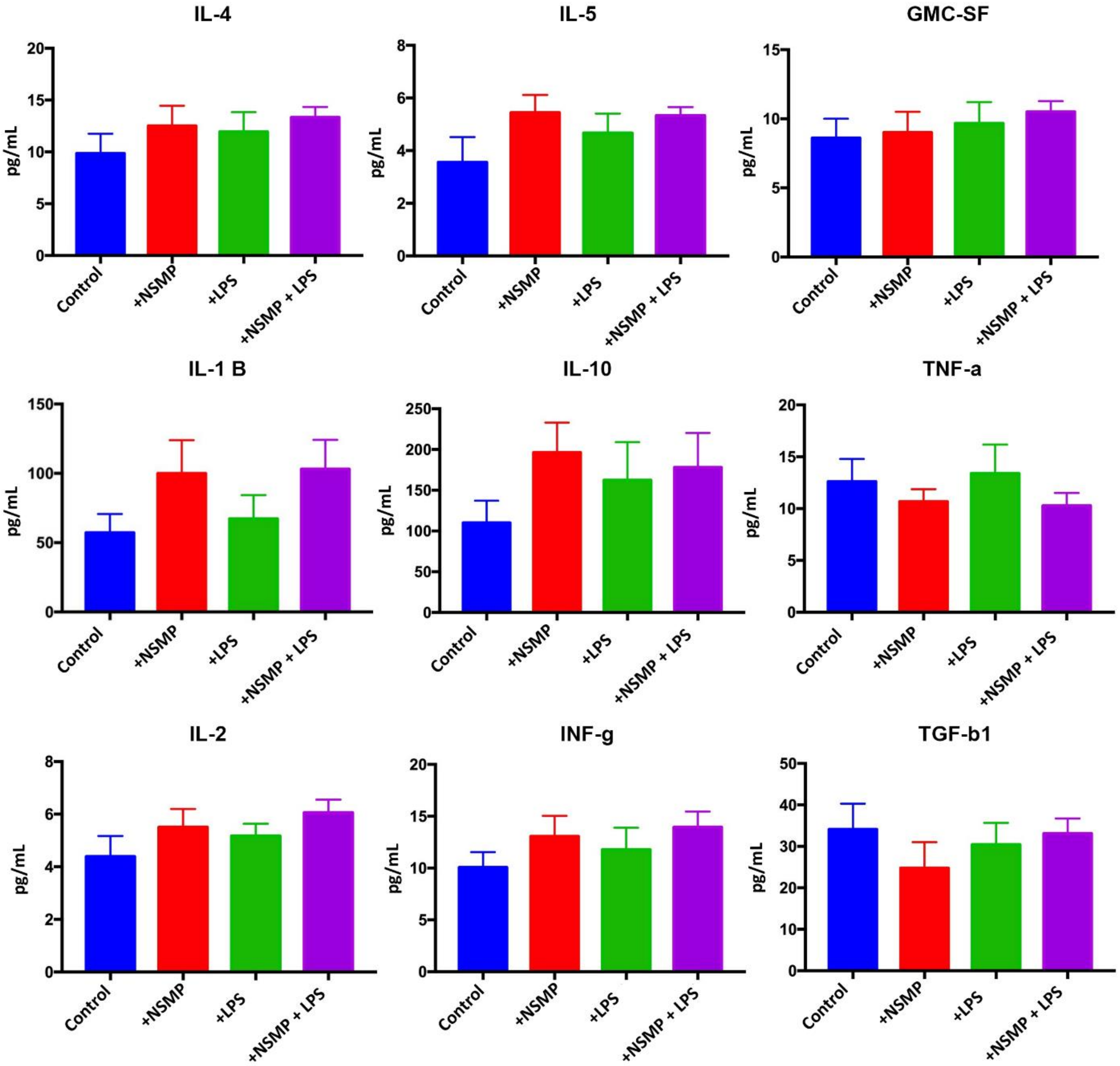

Supplementary Figure S2. Changes in the cytokine cascade when co-cultured with macrophages.
